# Reducing Pre‐screening Cognitive Assessment Variability to improve Enrollment in clinical trials

**DOI:** 10.1002/alz.089976

**Published:** 2025-01-09

**Authors:** Nicholas Toll

**Affiliations:** ^1^ JEM ‐ Headlands Reserach, Atlantis, FL USA

## Abstract

**Background:**

A standard practice at our research site has been the administration by a clinician of cognitive pre‐screeners to the subjects in our initial visit. The administration of these assessments, along with the examination of medical histories, is designed to evaluate a patient’s current cognitive abilities and assist with their placement in the most appropriate study. However, as the number of clinicians increased, variation regarding administration of the cognitive screener was noted, resulting in a likelihood of increased rate of screen failures.

**Method:**

To reduce the rate of screen failures at our research site and to increase the specificity of pre‐screening we implemented an improvement process to have one trained individual administering all of the standard cognitive pre‐screenings.

**Result:**

This design resulted in a 25% reduction of outcomes variability due to multiple examiners, as unique responses are more likely to be interpreted in a similar manner. The methodology reduced practice effects as cognitive screening questions may be inadvertently discussed prior to the screener (e.g., month, date, place) during interview with the subject and caregiver. Additionally, the process eliminates any potential for relationship bias, as the trained individual has no prior relationship to the subject, as well as assist the clinician in tailoring the interview to specific qualifying studies.

**Conclusion:**

The improvement process reduced the number of screen failure allowing our site to take another step in the long and difficult collaborative process involved with the creation and approval of novel medications.

